# Current News

**Published:** 1893-04

**Authors:** 


### Current News.

### THE ELEVENTH INTERNATIONAL CONGRESS OF MEDICINE.

The inauguration of the Eleventh International Congress of Medicine, to be held in Rome, September 24 to October 1, 1893, will take place the 24th of September, 1893, in the presence of His Majesty the King of Italy.

President, Professor G. Baccelli, Rome; Secretary General, Professor E. Maragliano, Genoa; Treasurer, Professor L. Pagliani, Rome.

The work of the Congress will begin in the nineteen Sections on the morning of the 25th of September. It will be continued in accordance with the arrangements to be made and published both for the general sessions and the Sections. Some of the general sessions will be devoted to scientific addresses delivered by scientists of all nations.

LIST OF THE SERIES.

1. Anatomy.

2. Physiology.

3. General Pathology and Pathological Anatomy.

4. Pharmacology.

5. Internal Medicine.

6. Diseases of Children.

7. Psychiatry, Neuropathology, and Criminal Anthropology.

8. Surgery and Orthopedy.

9. Obstetrics and Gynæcology.

10. Laryngology.

11. Otology.

12. Ophthalmology.

13. Odontology.

14. Military Medicine and Surgery.

15. Hygiene.

16. Sanitary Engineering.

17. Dermatology and Syphilidology.

18. Forensic Medicine.

19. Hydrology and Climatology.

REGULATIONS.

1. The Eleventh International Congress of Medicine will be inaugurated in Rome on the 24th of September, 1893, and will close on the 1st of October.

2. Any physician may become an active member of the Congress by fulfilling the conditions of membership, inscribing his name, and securing his admission ticket.

3. Scientists of other professions who, through their special studies, are interested in the labors of the Congress, may acquire the rights and assume the duties of active members, and participate in the work of the Congress, both by communications and discussions.

4. The fee for admission to the Congress is twenty-five francs, or five dollars.[1] It entitles to a copy of the Transactions of the Congress, which will be forwarded to the members immediately after publication.

---

[1] Money-order or check to the Treasurer, Professor Comm. L. Pagliani, Rome, Italy.

5. The character of the Congress is strictly and exclusively scientific.

6. The work of the Congress will be divided among nineteen Sections; every member is requested to indicate, on paying his admission-fee, the Section for which he desires to be inscribed.

7. The provisional committee will arrange the appointment, in the opening session, of the permanent officers. They will be a president, three vice-presidents, a number of honorary presidents, and secretaries. Each Section will elect, in its first meeting, its president and a certain number of honorary presidents, who shall alternately take the chair during the session. Some of the secretaries will be chosen from among the foreign members in order to facilitate the recording both of communications and of discussions in the different languages.

8. There will be daily sessions, either general or sectional. The times and numbers of the general sessions, and the business to be transacted in them, will be arranged by the President of the Congress.

9. The general sessions are reserved (*a*) for the consideration of the common work of the Congress and of its common interests; (*b*) for addresses and communications of general interest and importance.

10. The addresses in the general sessions, and in such extraordinary sessions as may be arranged, will be delivered by members chosen by the committee for the purpose.

11. Papers for and communications to the Congress must be announced on or before June 30, 1893. A brief abstract of every paper and communication, with their conclusions, must be sent to the committee on or before July 31. All of them will be printed and distributed to the members by authority of the President. Such as arrive after that date cannot be expected to find a place on the regular order of business, and will be accepted only if time will permit.

12. The business of the Sections will be arranged by their Presidents, who will also determine the hours of meeting, avoiding those reserved for the general sessions. Two or more Sections may hold joint meetings with the consent of their Presidents. There will be no vote on scientific questions.

13. Fifteen minutes are allowed for the reading of a paper or communication. In the discussion every speaker can have the floor but once, and for five minutes only. To close the discussion the author of the paper is allowed ten minutes. Additional time may be given him by the President, by special resolution of the Section, if the importance of the subject under discussion appears to require it.

14. The manuscript of all addresses, papers, and communications read either before a general session or a Section must be handed to the Secretary before the close of the meeting. A special Committee on Publication, appointed by the President, will decide which or what part of them shall be published in the Transactions of the Congress. Such members as participated in the discussions are required to hand to the Secretaries their remarks in writing.

15. The official languages of the sessions are Italian, French, English, and German. The regulations, programmes, and daily bulletins will be published in the above four languages. During the meetings, however, a member may be permitted to use, for a brief remark, any other language, provided some member present express his willingness to translate such remarks into any of the official languages.

16. The President directs the discussions according to the parliamentary rules generally obeyed in similar assemblies.

17. Persons not classified under Article 3, who are interested in the labors of a special Section, may be admitted by the President of the Congress. They will receive a special ticket on paying their admission fee; will not be entitled to a copy of the Transactions; and cannot speak in the general sessions, nor in any Section other than that for which they were inscribed.

18. The President may invite or admit students of medicine to attend and to listen. They will be given a special admission ticket, free of charge.

### GENERAL INFORMATION.

*Journeys and Reduction of Fares.*—The provisional committee has made arrangements with the different Italian and foreign railways and navigation companies, in pursuance whereof special reduced prices have been granted on the steamers and railways of this country and of the countries which the members of the Congress are to traverse.

In Italy the members of Congress will find tickets for round trips, starting from Rome; they will thereby be enabled to visit the most important cities and the various universities. In regard to this further notice will be given.

*The Ladies of the Members* will be furnished ladies' tickets, which will entitle them to the reduced fares granted to the members, and to participate in the festivities connected with the Congress.

*Festivities.*—Besides the receptions which the kind and hospitable citizens of Rome will offer to the members, the Italian colleagues will endeavor to return, to the best of their power, the kindness they experienced during their stay abroad.

On some evening, yet to be decided, the members of the different Sections will join at a dinner which will be given in one of the first hotels of Rome.

The Italian physicians have formed special committees to show the most hearty and kindly hospitality towards the foreign colleagues.

*International Exhibition of Medicine and Hygiene.*—On the occasion of the Eleventh International Medical Congress an Exhibition of Medicine and Hygiene will be inaugurated in Rome, which will gather all that may practically interest physicians and specialists. A special committee has already insured the co-operation of all the most important manufacturers of the world.

*Hotels.*—All the first- and second-class hotels of the Italian capital will afford to the members, during their stay, all desirable comforts.

---

### HARVARD ODONTOLOGICAL SOCIETY—ANNUAL MEETING.

THE Fifteenth Annual Meeting of the Harvard Odontological Society was held at Young's Hotel, Boston, Saturday, February 25, 1893, at 5.30 P.M., the President, Dr. Jere. E. Stanton, in the chair.

After the regular business had been transacted, the reports of the Recording Secretary, Treasurer, and Editor were read and accepted. Adjourning to the banquet-hall, forty-one members and invited guests did justice to the dinner set before them, and then gave their attention to the orator of the evening, Dr. Forrest G. Eddy, of Providence, R. I.

The subject of Dr. Eddy's paper was "Character in Professional Life," and he showed the advance dentistry had made during the past fifty years, and contrasted the dentist of the past, "who savored of the stable as well as the drugs of his office," with the truly professional man of to-day, whose recognized high standing was due chiefly to his character.

Dr. Dwight M. Clapp, as chairman of the Committee on the Harvard Dental School, reported that fifteen thousand dollars had already been subscribed in addition to the three thousand dollars promised by the alumni. He felt confident that the desired amount would be obtained, although it would not be raised in a day.

Letters of regret had been received from Hon. John Q. A. Brackett, ex-governor of Massachusetts, Rev. Edward A. Horton, General Charles H. Taylor, and Mr. S. Albert Wetmore, who had expected to be present.

In introducing the speakers of the evening President Stanton said that at our other meetings we devoted ourselves to subjects connected with dentistry, but at our annual dinner we invited those who were outside of our ranks, so that we might keep in step with the world.

The members of the Society then had the pleasure of listening to Rev. Dr. Moxom, Carl W. Ernst, A.M., assistant postmaster; Stephen O'Meara, A.M., of the *Boston Journal;* Rev. Dr. Green, of Providence, R. I.; David Hunt, M.D., and Samuel M. Child, Esq., who not only entertained them with bright remarks and witty stories, but also offered many suggestions of the most practical value. The great truth was forcibly brought out that while every profession is working in its own individual line, each apparently separate from the other, yet when we get nearest to the great object for which we are all striving,—the great good of humanity,—we find we have risen to a common level where we can touch elbows.

After the guests had departed, Dr. J. C. Ottinger, representing the Union Electric Works, of Chicago, Illinois, exhibited "The Crowdus Chemical Dental Outfit," which includes a battery, motor, drill, plugger, root-dryer, and incandescent mouth-lamp.

The Society then elected the following-named officers for the ensuing year:

President, Forrest G. Eddy, D.M.D., Providence, R. I.; Recording Secretary, Waldo E. Boardman, D.M.D., Boston; Corresponding Secretary, James Shepherd, D.M.D., Boston; Treasurer, Dwight M. Clapp, D.M.D., Boston; Editor, Henry L. Upham, D.M.D., Boston.

*Executive Committee.*—Waldo E. Boardman, D.M.D., Boston; Washburn E. Page, D.M.D., Boston; Jere. E. Stanton, M.D., D.M.D., Boston.

*Consultation Committee.*—Jere. E. Stanton, M.D., D.M.D., Boston; Dwight M. Clapp, D.M.D., Boston; Eugene H. Smith, D.M.D., Boston.

George F. Grant, D.M.D., Boston, was elected orator for 1894.

JAMES SHEPHERD,
*Corresponding Secretary.*

### NORTH CAROLINA STATE DENTAL SOCIETY.

MEMBERS OF THE NORTH CAROLINA DENTAL SOCIETY:

Your Executive Committee met in Raleigh at the call of the Chairman, and made out the following schedule, which, we believe, will be an effective one, and we heartily trust that there will be an earnest co-operation of the members to make this the best meeting in the history of the Society.

The Executive Committee finds that the date of meeting will have to be changed to the fourth Tuesday (23d) in May, that we may not conflict with the Georgia Association, which meets second Tuesday.

### STANDING COMMITTEES, 1893.

*Dental Education.*—J. E. Freeland, Statesville, N. C., Chairman.

*Dental Chemistry and Metallurgy.*—I. N. Carr, Tarborough, Chairman.

*Dental Pathology.*—J. H. Durham, Wilmington, Chairman.

*Dental Therapeutics.*—S. P. Hilliard, Rocky Mount, Chairman.

*Operative Dentistry.*—J. E. Matthews, Wilmington, Chairman.

Under this section the following special topics will be presented:

1. Should immediate root-filling be practised while purulent conditions exist at the apex? Dr. Matthews.

2. What are the best materials to enter into the composition of temporary fillings to be retained for a minimum of three years? Dr. Wyche.

3. What are the best methods of obtunding sensibility of the dentine, by either local or general means? Should arsenic ever be used? Dr. Wright.

*Prosthetic Dentistry, including Crown- and Bridge-Work.*—N. M. Culbreth, Whiteville, Chairman.

1. What are the best forms of partial lower dentures, and the methods of constructing the same? Dr. Culbreth.

2. Taking impressions. Dr. Hunter.

3. The Marshall anchor-plate and partial plates in general. Dr. London.

4. Crown- and bridge-work. Dr. Alexander.

*Orthodontia.*—J. F. Griffith, Salisbury, Chairman.

*Oral Surgery.*—W. H. Hoffman, Charlotte, Chairman.

*Materials and Appliances.*—J. W. Holt, Goldsborough, Chairman.

*Dental Prophylaxis.*—J. F. Ramsay, Asheville, Chairman.

F. S. HARRIS,
*For the Executive Committee.*

### COMMENCEMENTS.

THE Commencement of the Pennsylvania College of Dental Surgery was held at the Academy of Music, Philadelphia, at 12 o'clock Tuesday, February 28, 1893.

The address to graduates was given by Professor Wilbur F. Litch.

| | |
|---|---|
| Matriculates | 197 |
| Absent | 2 |
| Total | 195 |
| Graduates | 23 |

The Commencement of the Philadelphia Dental Collage took place at the college amphitheatre Wednesday, March 8, 1893, at 8 P.M.

The address to graduates was made by Professor H. I. Dorr, M.D., D.D.S.

The number of students in each of the three classes is as follows:

| | | |
|---|---|---|
| Freshmen | | 129 |
| Not in full attendance | 7 | |
| Deceased | 1 | |
| | | 8 |
| Total | | 121 |
| Juniors | | 79 |
| Not in full attendance | 2 | |
| Deceased | 1 | |
| | | 3 |
| Total | | 76 |
| Graduates | | 25 |

The Kansas City Dental College Commencement was held at the Coates House, Friday evening, March 3, 1893.

The Faculty address was given by Professor Charles H. Lester.

| | |
|---|---|
| Number of graduates | 4 |

